# Anatomical consideration for botulinum toxin injection of the frontalis muscle based on analysis of intramuscular innervation

**DOI:** 10.1038/s41598-025-93900-x

**Published:** 2025-03-14

**Authors:** Ju Eun Han, Taeyeon Kim, Shin Hyo Lee, Kang-Jae Shin

**Affiliations:** 1 Department of Anatomy and Cell Biology, Dong-A University College of Medicine, 32 Daesingongwon-ro, Seo-gu, Busan, 49201 Republic of Korea; 2 Translational Laboratory for Clinical Anatomy, Department of Anatomy, Yonsei University College of Medicine, Seoul, Republic of Korea; 3 Department of Anatomy, Wonkwang University School of Medicine, Iksan, Republic of Korea; 4 Jesaeng-Euise Clinical Anatomy Center, Wonkwang University School of Medicine, Iksan, Republic of Korea

**Keywords:** Temporal branch of facial nerve, Frontalis muscle, Botulinum toxin, Sihler’s staining, Facial palsy, Anatomy, Medical research

## Abstract

The facial nerve is the seventh cranial nerve, and its temporal branch (TBFN) innervates the frontalis muscle. Peripheral nerve disorders involving the facial nerve can lead to facial palsy, for which a common non-invasive treatment approach is to inject a chemodenervation agent such as botulinum toxin (BoNT). The purpose of this study was to provide anatomical suggestions for BoNT injection sites in the frontalis muscle based on the intramuscular innervation pattern of the TBFN as identified objectively using Sihler’s staining. Nineteen hemifaces containing the TBFN and the frontalis muscle were harvested from 15 embalmed cadavers according to facial landmarks. The frontalis muscle was divided into 16 areas to identify the prevalence rates of distal nerve endings and the arborization pattern of the TBFN after applying Sihler’s staining. Distal nerve endings of the TBFN were most commonly found in area B2 (17 of 19 specimens, 89.5%), followed by in area B3 (*n* = 15, 78.9%). No distal nerve ending was observed in area A1. Two types of the arborization pattern of the TBFN were observed. We propose four BoNT injection sites based on the intramuscular innervation pattern of the TBFN in the frontalis muscle as identified using Sihler’s staining.

## Introduction

The facial nerve (the seventh cranial nerve) is a mixed nerve comprising motor, sensory and parasympathetic fibers, and its motor fibers innervate the muscles that control facial expressions. After leaving the brainstem, the nerve runs in the temporal bone along the facial canal and exits the intracranial part through the stylomastoid foramen. Forming the parotid plexus, it is divided into five terminal branches: temporal, zygomatic, buccal, marginal mandibular, and cervical branches. The temporal branch of the facial nerve (TBFN) crosses the zygomatic arch to the temporal region and passes through the areolar tissue between the superficial and deep temporal fasciae^1–3^. The frontalis muscle innervated by the TBFN originates from the galea aponeurotica, which corresponds with the hairline on the skull surface, and covers almost the entire forehead. The muscle influences the shape and position of the eyebrows by elevating them.

Facial palsy is a peripheral nerve disorder involving the facial nerve that is caused by idiopathic conditions or various neuropathic conditions such as infection and trauma. The reported annual incidence of facial palsy is 15–30 per 100,000 persons, with equal numbers of males and females affected, and it occurs in patients of all ages, with the incidence peaking at 40–49 years of age^4^. The severity of aesthetic changes is related to the absence of movement on the paralyzed side, and also to the response of the mimetic musculature after the loss of balance between the paralyzed and moving sides. The compensatory hyperactivity of the frontalis muscle on the non-paralyzed side, acting against the weak antagonism of the contralateral muscles, leads to the appearance of wrinkles and furrows over the frontalis muscle along with asymmetry of the eyebrow position^3,5,6^. Various treatment methods are applied (e.g., oral corticosteroids or antiviral drugs, or even simply spontaneous recovery) depending on the cause of facial palsy, with non-surgical treatment being used more than surgical treatment^7^.

The injection of a chemodenervation agent such as botulinum toxin (BoNT) is currently the most commonly performed non-invasive procedure for facial palsy on the upper face^8,9^. BoNT has been used since 1987 for treating asymmetries caused by facial paralysis on the non-paralyzed side and is injected into targeted muscles on the unaffected side to reduce hyperkinesis, which results in significant facial aesthetic improvements. Furthermore, the effect of BoNT in controlling the excessive movement of facial expression muscles is well known and used as a global protocol for facial rejuvenation^8,10^.

BoNT blocks acetylcholine release at the neuromuscular junction, which leads to reversible muscle paralysis without long-term damage to the muscle or nerve^5^. The outcome of injecting BoNT varies according to the amount of toxin that reaches the distal nerve ending, and so it is recommended that the injection targets the adjacent peripheral nerve endings^11^. It is essential that physicians conducting BoNT injections have adequate anatomical knowledge of intramuscular nerve innervation^5,9^. Although some previous studies have conducted whole-face Sihler staining, there is limited information regarding the intramuscular innervation pattern of the frontalis muscle, particularly focusing on the TBFN^12–15^.

The aim of this study was to provide anatomical suggestions for BoNT injection sites in the frontalis muscle based on the intramuscular innervation of the TBFN using Sihler’s staining, a whole-mount staining technique.

## Materials & methods

Nineteen hemifaces from 15 embalmed cadavers (12 male and 3 female) were dissected and subjected to Sihler’s staining to elucidate the intramuscular innervation of the frontalis muscle. The mean age at the time of death was 76.3 years. We obtained appropriate approval from the Institutional Review Board (IRB) of Dong-A University for the use of cadavers as well as for the experimental protocol used in the present study (IRB No. 2-1040709-AB-N-01-202305-BR-003-02). Before they died, each donor signed documents agreeing to participate in the body donation program of Dong-A University College of Medicine and to allow the use of their body for clinical studies. This study was conducted in accordance with the principles outlined in the Declaration of Helsinki. The cause of death of the 15 cadavers used in the present study was natural aging and cancers. These causes of death did not affect the hemiface specimens. The specimens included had no gross pathology or signs of surgical procedures in the forehead, eyelids, and temporal region.

### Specimen harvesting

**Fig. 1:**
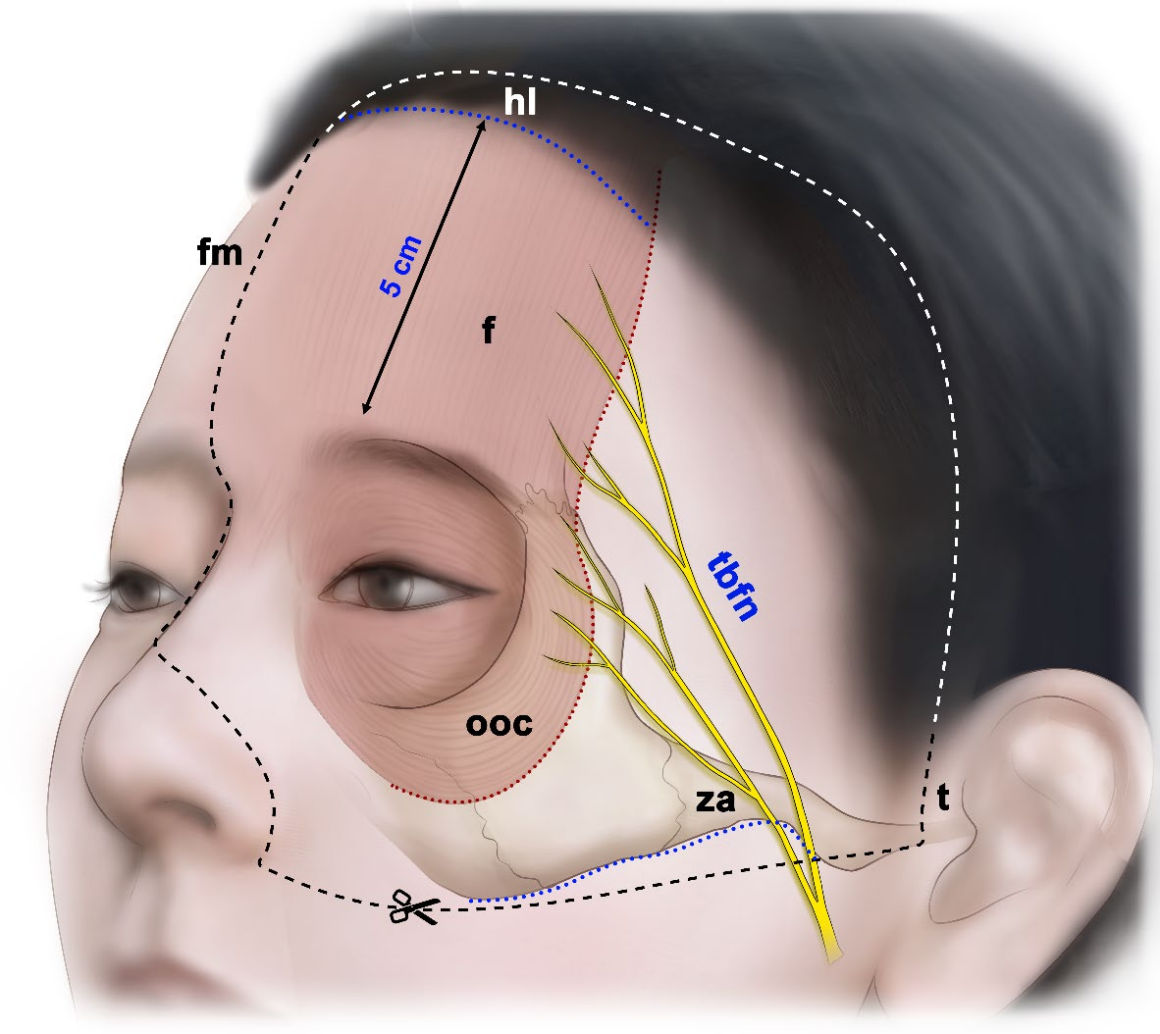
Incision lines for specimen harvesting. Nineteen hemiface specimens including the frontalis and orbicularis oculi muscles and the facial nerve were detached from the skull. hl, hairline; fm, facial midline; t, tragion; za, zygomatic arch; tbfn; temporal branch of facial nerve; f, frontalis muscle; ooc, orbicularis oculi muscle.

The skin and subcutaneous tissue of the upper and midface were removed except for the eyebrows, and the margins of the frontalis and orbicularis oculi muscles were delineated. Hemifaces that included the frontalis and orbicularis oculi muscles and the eyebrow were harvested with the following four boundaries: a horizontal line 5 cm above the eyebrows (hairline), a horizontal line along the inferior border of the zygomatic arch, a vertical line passing through the tragion, and a vertical line along the facial midline (Fig. 1).

### Sihler’s staining

We used Sihler’s staining, which is a whole-mount nerve staining technique that renders soft tissue translucent or transparent while staining the peripheral nerve axons^9,16^. The procedure was carried out according to the Sihler’s staining protocol previous studies^17,18^. The Sihler’s staining method applied in this study consisted of seven steps, and it was most important to make frequent observations and replace reagents in a timely manner during the staining step. The timing of the staining process differed slightly between samples depending on the thickness or degree of adipose tissue.

The following staining steps were used in this study:

*Fixation* The hemiface specimens were first fixed in 10% non-neutralized formalin for 2 ~ 4 weeks. The fixative solution must be replaced whenever it cloudy.

*Maceration and depigmentation* The fixed specimens were cleaned in running tap water for 1 h and then placed in a maceration solution (3% aqueous potassium hydroxide solution containing 0.2 ml of 3% hydrogen peroxide per 100 ml) for 4 weeks. This solution should be changed every 1–2 days until the specimen becomes well bleached and translucent.

*Decalcification* The specimens were then decalcified for 4 weeks in Sihler’s solution I (glacial acetic acid: glycerin:1% aqueous chloral hydrate at 1:1:6). The decalcifying solution was changed once a week throughout the process.

*Staining* Thereafter the muscles were stained for 2 ~ 4 weeks in Sihler’s solution II (Ehrlich’s hematoxylin: glycerin:1% aqueous chloral hydrate at 1:1:6) until it stained deep purple. The staining condition, which is the criterion that determines progress to the next step, can be evaluated by observing the degree of penetration by Ehrlich’s hematoxylin on a viewing box with sufficient light transmission.

*Destaining* The nerves were made visible by resoaking in Sihler’s solution I for 3–4 h with occational agitation. Generally, this process is stopped when the stained nerve twigs become visible. However, in the present study, the destaining time was reduced to 3–4 h to better visualize the fine nerve branches distributed in the frontalis muscle.

*Neutralization* The specimen, which at this stage is in an acidic state, needs to be neutralized by soaking in 0.05% lithium carbonate solution for approximately 1 h and then washed under running tap water for 1 h.

*Clearing* Finally, the frontalis muscles were displayed by soaking them in a series of glycerin solutions of increasing concentrations (40%, 60%, 80%, and 100%). The specimens were left in each solution for 1 day or until the excess stain was cleared out. The specimens were then stored in pure glycerin.

**Fig. 2:**
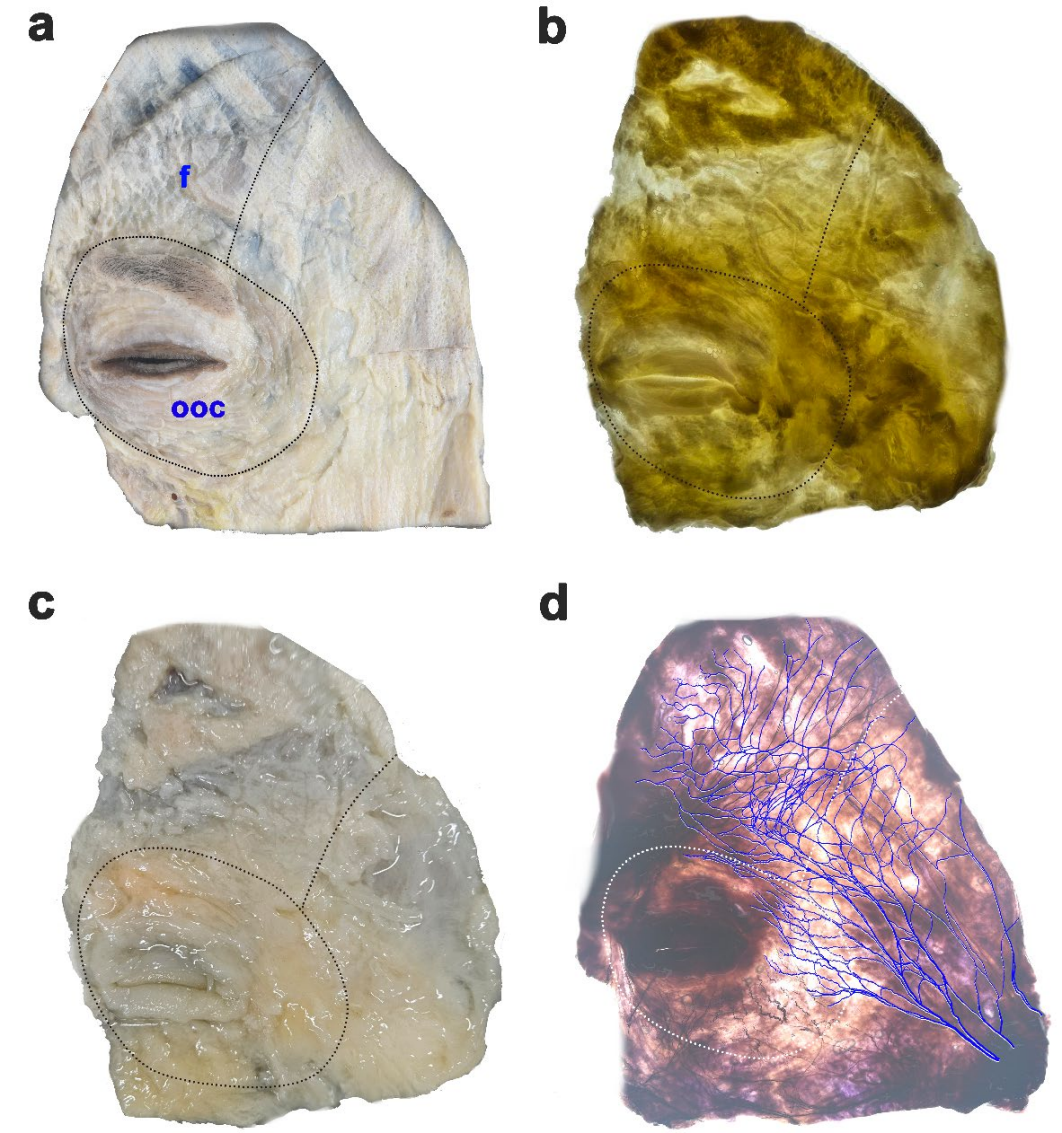
Specimens in different steps of Sihler’s staining: fixation (dashed lines, margins of the frontalis and orbicularis oculi muscles) (**a**); maceration and depigmentation (**b**); decalcification (**c**) and clearing (blue tracks, temporal branch of facial nerve) (**d**).

After applying Sihler’s staining, the intramuscular innervation of the frontalis muscle was observed on a viewing box, and fine twigs of the TBFN were carefully dissected under 3× loupe magnification (Fig. 2).

### Intramuscular innervation pattern of the frontalis muscle

**Fig. 3:**
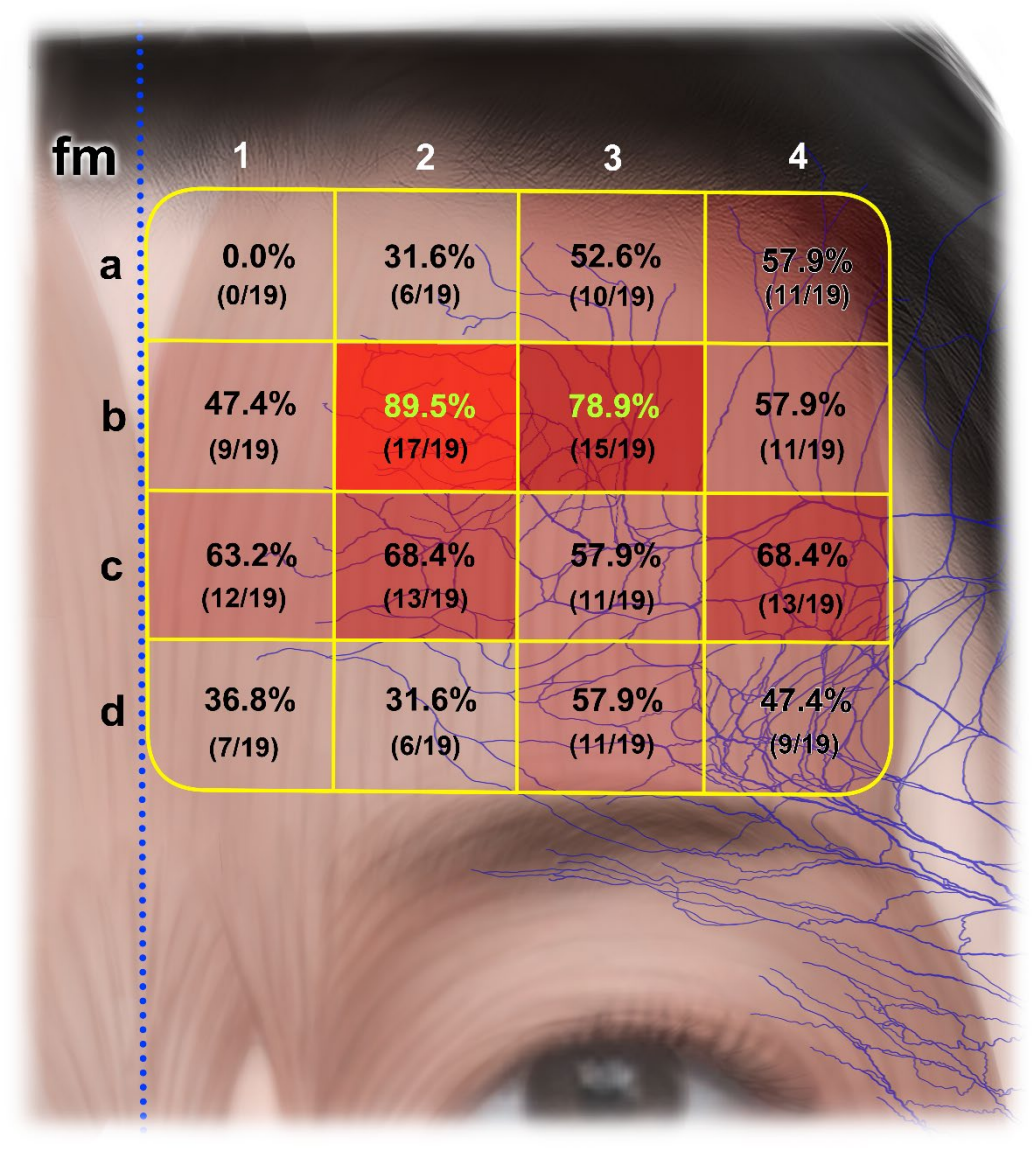
Prevalence rates of distal nerve endings in the stained frontalis muscle specimens.

The frontalis muscle was divided into four rows transversely above the superior margin of the eyebrow and into four columns laterally from the facial midline, with the horizontally divided areas labeled numerically and the vertically divided areas labeled alphabetically (Fig. 3). We determined whether distal nerve endings were present in each of the resulting 16 areas of the frontalis muscle.

## Results

The presence of distal nerve endings in the 16 areas of the frontalis muscle was expressed as a percentage of the total number of specimens. Distal nerve endings of the TBFN were most commonly located in area B2 in 17 of the 19 specimens (89.5%), followed by in area B3 in 15 specimens (78.9%), in areas C2 and C4 in 13 specimens (68.4%), in area C1 in 12 specimens (63.2%), and in areas C3, D3, A4, and B4 in 11 specimens (57.9%). No distal nerve ending was observed in area A1 (Fig. 3).

**Fig. 4:**
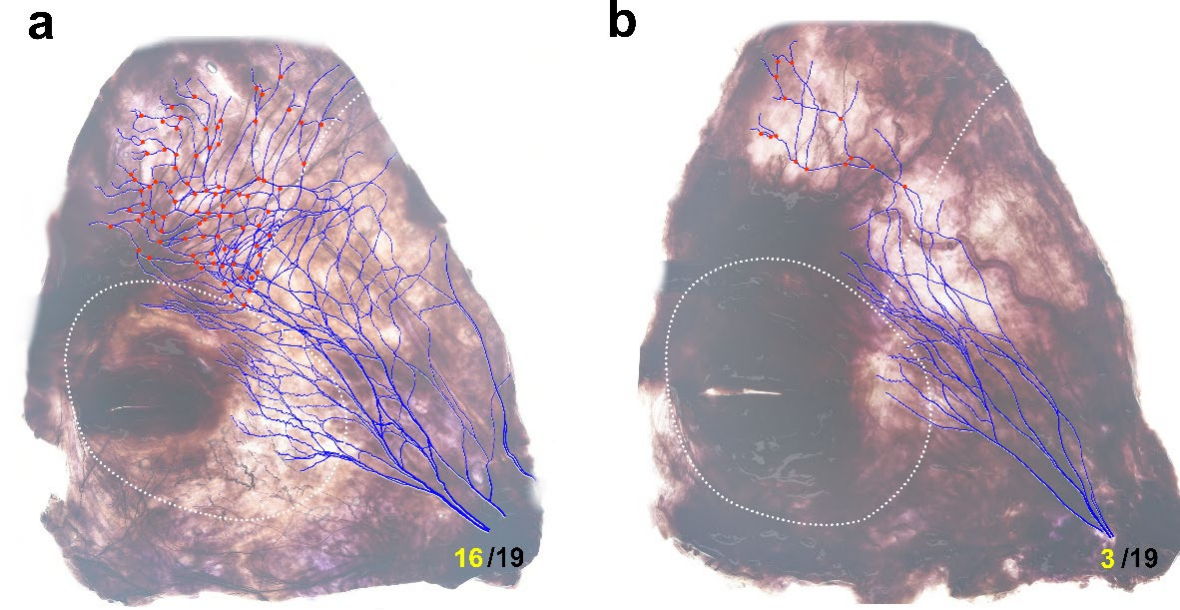
Two types of the intramuscular arborization pattern of the TBFN: typical type (16/19, 84.2%) (**a**) and atypical type (3/19, 15.8%) (**b**). Red dots indicate the locations of neural branching.

Two types of the arborization pattern of the TBFN were observed. Three out of the 19 specimens (15.7%) showed a markedly low number of the nerve braches (Fig. 4).

## Discussion

Injecting BoNT is a widely utilized procedure in various clinical fields, including the restoration of facial imbalance caused by facial palsy and facial rejuvenation through the reduction of skin wrinkles^8,10,11,19,20^. do Nascimento Remigio et al. demonstrated a sustained 9% reduction in facial asymmetry at 6 months after injecting BoNT into the non-paralyzed side of the face^21^. There are numerous guidelines for injecting BoNT into the frontalis muscle based on clinical experiences. Small volumes of BoNT solution are typically injected into from three to nine sites, with doses ranging from 0.5 to 2 U per site. These injections are performed using a 30-gauge, 1-inch needle, which produces a halo of action with a radius of 1.0 ~ 1.5 cm. Progress is often monitored from 1 to 6 months postinjection^8,19,21^. The injection technique involves inserting the needle while it is angled 30% upward and extending the injection sites laterally along the lateral canthus^22^. Since the inferior portion of the frontalis muscle intersects with muscle fibers of the procerus and orbicularis oculi muscles, to avoid unexpected complications the recent guidelines suggest that the injections should be performed 2.5–3 cm above the superior margin of the orbit or 1–2 cm above the superior margin of the eyebrow^6,12,19,23^. Another previous study supported that the upper limit of the injection is 1 cm below the hairline, which corresponds to the level of the galea aponeurotica^19^. Despite numerous clinical suggestions, there is a lack of basic anatomical evidence such as about the intramuscular innervation of the frontalis muscle for determining the optimal site of BoNT injection, and there are also no consent guidelines for its implementation in facial palsy^21^.

Common side effects of BoNT injections into the frontalis muscle include eyebrow ptosis, an elevated appearance of the eyebrow tail, eyebrow asymmetry, and diplopia^12,22,24^. These are technique-dependent complications that may result from the diversity of intramuscular innervation patterns of the frontalis muscle among patients. The appearance of a ‘Mephisto sign’ (‘samurai eyebrow’) can vary with the length, structure, and morphology of the frontalis muscle^24,25^. To prevent the Mephisto sign, previous authors have recommended performing an additional injection of BoNT into the lateral end of the frontalis muscle, restricted to the area between the midpupillary lines^12^. This recommendation could be explained by the present findings. Because areas A4, B4, and C4 showed relatively high prevalence rates of distal nerve endings (57.9%, 57.9% and 68.4%, respectively), overlooking these areas may result in the Mephisto sign appearing due to compensatory hyperactivity of the lateral end of the frontalis muscle. The shape and size of muscles might also not be the only factors influencing this complication, with the intramuscular innervation pattern (locations of distal nerve endings) possibly also contributing to the Mephisto sign^6,22^.

It has been recommended to inject into the medial part of the frontalis muscle at 1.5–2.0 cm from the facial midline^12^. This is based on the morphology of the frontalis muscle, which has a V-shaped appearance with a slight depression between the bilateral muscles due to midline dehiscence (approximately 3.5 cm above the eyebrow arch). Consequently, administering BoNT at the midline of the forehead may be unnecessary or may require only a minimal amount. This is theoretically supported by our findings of distal nerve endings not being present in area A1 and being present in area B1 in less than half of the specimens. The two sides of the frontalis muscle intersect in area C1, and the angulation between these muscles was more acute in females than in males. Therefore, injecting into area C1 could be expected to be effective in females^26^. On the other hand, injections into area D1 should be restricted to avoid unexpected complications resulting from the frontalis muscle overlapping the procerus, corrugator supercilii, and orbicularis oculi muscles in that area^12^.

Previous studies have recommended that the upper limit for injections should be about 1 cm below the hairline due to presence of the galea aponeurotica, with the lower limit being either 2.5–3.0 cm from the superior orbital margin or 1.5–2.0 cm above the eyebrow due to the presence of the orbicularis oculi muscle^12,27^. These clinical recommendations were suggested based on the structural relationships of facial muscles, and they could also be supported by our findings. Distal nerve endings were more prevalent in rows B and C than in rows A and D: there were no distal nerve endings in area A1 in any specimen, and their prevalence rates were low in areas A2, D1, D2, and D4 (31.6–47.4%). Although area D3 showed a relatively high prevalence of distal nerve endings (57.9%), consideration of the structural relationships of the facial muscles may mean that the identified nerve endings were for the orbicularis oculi muscle rather than for the frontalis muscle^25^. Therefore, BoNT injections should not be performed in the uppermost and lowermost areas (rows A and D) due to possibility of side effects and also their low effectiveness.

The central areas in the present study (B2, B3, C2, and C3) are the most-common and well-known injection sites for the frontalis muscle, and presented the highest prevalence rates of distal nerve endings (57.9–89.5%). As references for injection, the midpupillary line is drawn and a site is marked about 2 cm above the superior margin of the eyebrow. Injections are then performed at three sites in a row, spaced approximately 2 cm apart on either side^6,8,19,23^. This 2-cm interval is based on the assumption that BoNT will diffuse approximately 1 cm from the injection site, since the toxin is usually diluted with saline to produce an injected volume of 2.0–3.3 ml. The injection sites of this three-site technique, similar to the central areas in the present study, were chosen because wrinkles are most prominent in these parts of the forehead and they do not overlap with other facial muscles^6,28^. The findings of the present study have also demonstrated that these injection sites may be most effective.

**Fig. 5:**
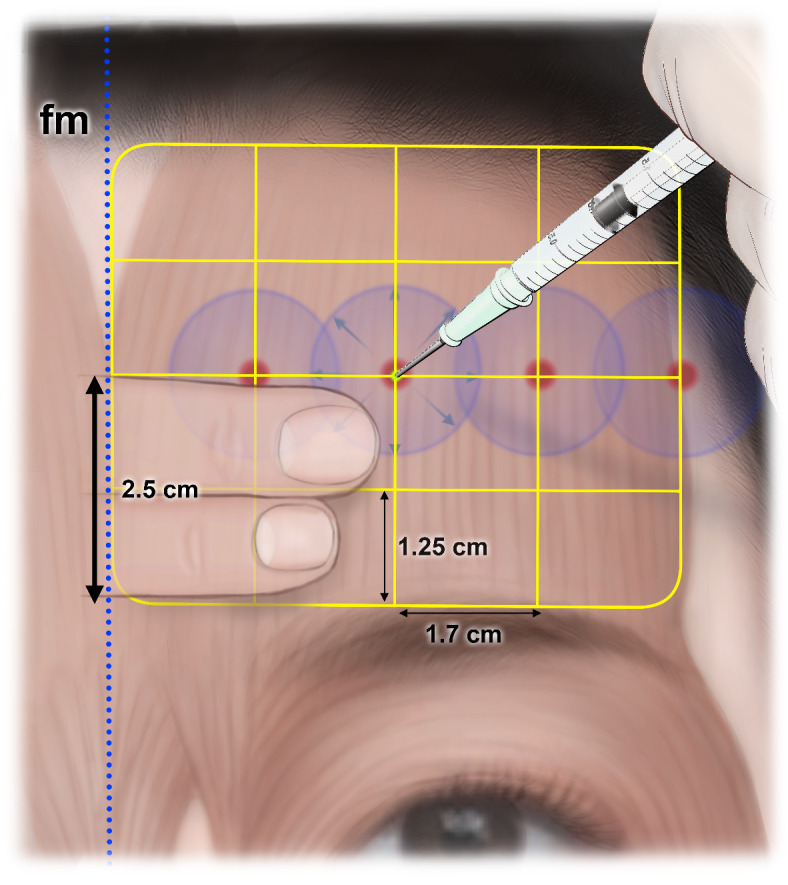
Four anatomical sites suggested for injecting botulinum toxin into the frontalis muscles based on the intramuscular innervation patterns.

From a comprehensive consideration of the previously reported information on injection techniques and the results of the present study that utilized objective Sihler’s staining, we propose four injection sites at the boundaries between rows B and C (Fig. 5). Although the dimensions of the forehead differ among individuals, the average forehead height (the vertical distance from the eyebrow to the hairline) and hemiforehead width (the horizontal distance between the facial midline to the frontotemporale) can be considered to be around 5 cm and 6–7 cm, respectively^27,29–31^. The present study divided the frontalis muscle on one side into four sets of areas vertically and four sets of rows horizontally. The height and width of each area were approximately 1.25 cm and 1.7 cm, respectively. The commonly injected volume of BoNT can diffuse approximately 1 cm from each injection site (2 cm in diameter), and so administering this at the four sites should result in it covering almost all of the areas with the highest prevalence rates of distal nerve endings whilst also avoiding complications such as the Mephisto sign and eyebrow ptosis. In addition, the physician can easily and rapidly identify these injection sites during the procedure because the distances from the eyebrow to the injection sites are approximately 2.5 cm (1.25 cm + 1.25 cm), allowing them to be measured using two (e.g., the second and third) fingers (Fig. 5).

Two types of TBFN arborization patterns were observed. The number of nerve branches in 3 specimens was markedly lower than those in the other 16 specimens (Fig. 4). The variability in the intramuscular innervation pattern of the TBFN could lead to individual differences in the effectiveness of the procedure even when applying the same BoNT injection technique. Physicians should therefore handle cases flexibly according to the symptoms of individual patients based on clear anatomical evidence such as the present research findings.

The main limitation of the present study was that we did not directly investigate the position of the frontalis muscle predictable on the facial surface. However, previous studies have already demonstrated palpable surface landmarks on the lateral border of the frontalis muscle. This border runs parallel to a line that extends at 45 degrees from the most-prominent part of the frontotemporal region, positioned 1 cm inward from this line^22,27,32^. Based on previous studies it is therefore possible to predict the lateral border of the frontalis muscle using the surface landmark. This allows physicians to determine the 16 areas on a patient’s forehead and use them to identify the optimal 4 injection sites as proposed by the present research.

In conclusion, the present study has objectively verified the scientific relevance of previously reported clinical guidelines for BoNT injections into the forehead and proposed four anatomical injection sites based on objective findings obtained from Sihler’s staining method. However, the present study has also identified that the effectiveness of the procedures could differ among patients even when using the same injection technique due to the variability in the arborization pattern of the TBFN. Moreover, BoNT formulations such as the diluent used (e.g., saline or lidocaine) and the injection volume and technique also vary among physicians^33,34^. Therefore, it is essential for physicians to consider variables such as symptoms, wrinkle locations, and typical facial expressions of individual patients based on our anatomical suggestions.

## Data Availability

The datasets generated and analysed during the current study are available from the corresponding author on reasonable request.
